# Effect of the Glycemic Index of Carbohydrates on *Acne vulgaris*

**DOI:** 10.3390/nu2101060

**Published:** 2010-10-18

**Authors:** Rebecca C. Reynolds, Stephen Lee, James Y. J. Choi, Fiona S. Atkinson, Karola S. Stockmann, Peter Petocz, Jennie C. Brand-Miller

**Affiliations:** 1 Human Nutrition Unit, School of Molecular Bioscience G08, The University of Sydney, NSW 2006, Australia; Email: fiona.atkinson@sydney.edu.au (F.S.A.); karola.stockmann@sydney.edu.au (K.S.S.); jennie.brandmiller@sydney.edu.au (J.C.B.M.); 2 Department of Dermatology, Concord Repatriation General Hospital, The University of Sydney, NSW 2006, Australia; Email: stephen.lee@sydney.edu.au (S.L.); jameschoi@optusnet.com.au (J.Y.J.C.); 3 Department of Statistics, Macquarie University, NSW 2109, Australia; Email: ppetocz@efs.mq.edu.au

**Keywords:** glycemic index, carbohydrate, diet, *acne vulgaris*, adolescent males

## Abstract

*Acne vulgaris* may be improved by dietary factors that increase insulin sensitivity. We hypothesized that a low-glycemic index diet would improve facial acne severity and insulin sensitivity. Fifty-eight adolescent males (mean age ± standard deviation 16.5 ± 1.0 y and body mass index 23.1 ± 3.5 kg/m^2^) were alternately allocated to high or low glycemic index diets. Severity of inflammatory lesions on the face, insulin sensitivity (homeostasis modeling assessment of insulin resistance), androgens and insulin-like growth factor-1 and its binding proteins were assessed at baseline and at eight weeks, a period corresponding to the school term. Forty-three subjects (n = 23 low glycemic index and n = 20 high glycemic index) completed the study. Diets differed significantly in glycemic index (mean ± standard error of the mean, low glycemic index 51 ± 1 *vs.* high glycemic index 61 ± 2, p = 0.0002), but not in macronutrient distribution or fiber content. Facial acne improved on both diets (low glycemic index −26 ± 6%, p = 0.0004 and high glycemic index −16 ± 7%, p = 0.01), but differences between diets did not reach significance. Change in insulin sensitivity was not different between diets (low glycemic index 0.2 ± 0.1 and high glycemic index 0.1 ± 0.1, p = 0.60) and did not correlate with change in acne severity (Pearson correlation r = −0.196, p = 0.244). Longer time frames, greater reductions in glycemic load or/and weight loss may be necessary to detect improvements in acne among adolescent boys.

## 1. Introduction

*Acne vulgaris* (acne) is a prevalent skin disorder with substantial physical and psychological morbidity. Although dietary factors have long been considered unimportant, insulin resistance (IR) and dietary carbohydrates have recently been implicated in the etiology of acne [1,2]. The glycemic index (GI) of meals has been directly correlated to insulin response [3] and low GI diets have been shown to decrease IR [4]. High insulin concentrations in the fasting and/or postprandial state may exacerbate acne by increasing the proliferation of basal keratinocytes. Insulin also stimulates the synthesis of androgens leading to high sebum production, a recognized correlate of acne severity [5]. IR could also increase inflammatory responses within and adjacent to the comedo [6].

Postprandial insulin responses may be of particular relevance during puberty and adolescence when whole-body IR naturally increases [7]. Insulin sensitivity and sex-hormone-binding globulin (SHBG) are directly correlated, such that falls in insulin sensitivity increase the free androgen index (FAI) [8,9]. Similarly, compensatory hyperinsulinemia is associated with reductions in insulin-like growth factor binding protein-1 (IGFBP-1), corresponding to higher cellular concentrations of free insulin-like growth factor-1 (IGF-1) [9]. Indeed, the highest incidence of acne occurs when IGF-1 levels peak [10] and adult women with acne have high levels of IGF-1 [11]. Interventions that reduce fasting and postprandial insulinemia and IGF-1 concentrations would therefore be expected to decrease sebum production and keratinocyte proliferation [12]. 

In the first study of its kind, Smith *et al.* demonstrated that reductions in carbohydrate intake with particular emphasis on low glycemic index (GI) carbohydrate foods, improved cutaneous and hormonal markers of acne severity in young adult males [2]. Several confounders, however, complicated the interpretation of the study because the treatment group lost more weight, and had higher protein and fiber intake than the control group—factors which the authors discussed. Since weight loss as well as higher protein and fiber intake have all been linked to improved insulin sensitivity, it is not clear whether dietary GI played an important role in the improvement of acne.

In the present study, we hypothesized that in the context of weight maintenance and identical macronutrient and fiber intake, the replacement of high GI carbohydrates with low GI carbohydrates would improve acne severity by lowering postprandial insulin concentrations and/or decreasing IR. We studied adolescent males attending boarding school so that food intakes could be more easily controlled. 

## 2. Methods

### 2.1. Subjects

Healthy adolescent males with acne were recruited at three secondary boarding schools in Sydney, Australia, between 2006 and 2007. Inclusion criteria were acne severity grade 1, 2 or 3, stable weight over the past three months and parental/guardian consent. Exclusion criteria were previous use of isotretinoin, antibiotics in the past month, excessive alcohol intake, illicit drug use, smoking, physical or mental illness, food allergy or intolerance, vegetarianism, previous surgery on the gastrointestinal system, black skin (due to difficulty in visualizing lesions), or final school examinations in the coming months. This study was conducted according to the guidelines laid down in the Declaration of Helsinki and all procedures involving human subjects were approved by the Sydney University Human Research Ethics Committee. Written informed consent was obtained from all subjects and their parents/guardians. The trial is registered with the Australian New Zealand Clinical Trials Registry (ANZCTR) ACTRN12610000261011 and Unique Trial Number (UTN) U1111-1114-5335.

### 2.2. Study protocol

The study was of parallel design and volunteers were assigned alternately to the high or low GI diet in order of recruitment. They were informed that the study was designed to determine the effect of the GI of carbohydrates on acne. Subjects were instructed to maintain their current washing regime. At baseline, height and weight were measured in light clothing and without shoes. Weight was recorded weekly and food diaries were completed on Saturday and Sunday of every week. Dietary records were analyzed using Food Works software package 3.02 (Xyris Software, Highgate Hill, Australia), based on the Australian food composition tables. Average weighted GIs were calculated from the diaries using the GI values of individual food components [13] and their relative contribution to total carbohydrate in the meal. At baseline and at eight weeks, severity of facial acne was assessed by a dermatologist. Blood (6 mL) was obtained via a cannula inserted into the antecubital vein of the lower arm and transferred into EDTA-coated Vacutainer tubes before being stored at −20 °C.

### 2.3. Diets

In one-to-one weekly counseling sessions, subjects were given detailed instruction on how to follow an *ad libitum* high or a low GI diet, with written material to supplement these sessions. Diet advice differed only in the type of carbohydrate to be eaten at meals provided in the school dining hall/at home. School menus were assessed to ensure high and low GI options were available at every meal, and instructions were sent to parents if boarders returned home at weekends. Snack foods were provided in hampers on a weekly basis. Low GI foods included verified low GI brands of muesli, a sports drink and a processed fruit snack, and lasagne [13]. High GI foods included verified high GI brands of wheat breakfast biscuits, a sports drink and a processed fruit snack, and baked potato with chili con carne [13].

### 2.4. Assessment of acne severity

A dermatologist (SL or JC) who was blind to diet allocation assessed facial acne severity by examining the number and degree of inflammation of inflammatory lesions (papules, pustules and nodules, not comedones or scars) on the face under ceiling fluorescent lighting or daylight. Acne was graded on a scale from 0 to 3 (0: no acne, 1: mild, 2: moderate and 3: severe). Photographs were taken of the forehead and right and left cheeks. When illness precluded the attendance of the first dermatologist (SL), a second dermatologist (JC) completed some of the final assessments. The first dermatologist later graded photographs from these assessments and an average was taken from the two grades of facial acne severity.

### 2.5. Blood analysis

Vacutainer tubes (6 mL) were centrifuged (Eppendorf 5702R refrigerated centrifuge) at 3000 rpm (112 RCF) for 5 min at 4 °C, plasma aliquoted and stored at −80 °C until assay. Glucose was measured in-house using a glucose hexokinase assay (Roche Diagnostics Corporation, Indianapolis, IN). Insulin was measured in-house in duplicate by competitive RIA (Coat-a-Count, Diagnostic Products, Los Angeles, CA, intra-assay CV 4.6%). Homeostasis modeling assessment of IR (HOMA-IR) was calculated as follows: glucose mmol/L × (insulin pmol/L/6)/22.5 [14]. Testosterone (total), SHBG, dihydroepiandrosterone-sulfate (DHEA-S) and total IGF-1 were measured externally at the Biochemistry Department of the Royal Prince Alfred Hospital, Sydney: testosterone and DHEA-S by competitive chemiluminescence EIA (Immulite 2000, Siemens Medical Solutions Diagnostic, Los Angeles, CA), SHBG by two-site chemiluminescence EIA (Immunite 2000, Siemens Medical Solutions Diagnostic, Los Angeles, CA) and IGF-1 by in-house RIA. IGFBP-1 and -3 were measured externally at the Molecular Medicine Department of the Royal North Shore Hospital by RIA (Baxter *et al.* [15] for IGFBP-1 and Baxter and Martin [16] for IGFBP-3). The FAI was calculated as: (testosterone nmol/L × 100)/SHBG nmol/L. 

### 2.6. Statistical analysis

Power calculations based on previous studies [17] showed at least 15 subjects per dietary intervention would provide >90% power to detect an effect size of ~50% in acne severity between the low and the high GI diets (p < 0.05). In the final analysis, a general linear model for change in acne severity was used, in which dermatologist and diet were fixed factors and baseline values were treated as a covariate (this allowed for the change in dermatologist as well as the change in the method of facial acne assessment, *i.e.*, photo *vs.* in-person). Changes over time within diets were compared using paired Student’s t-tests. Differences between the degree of change on each diet were compared using independent samples Student’s t-tests. Pearson’s correlations between acne severity and biochemical parameters were obtained. Statistical calculations were carried out using SPSS 14 for Windows (SPSS Inc., Chicago, IL). Results are expressed as mean ± SEM unless otherwise stated. Differences are considered significant if p < 0.05 and highly significant if p < 0.01.

## 3. Results and Discussion

A total of 58 subjects met the inclusion criteria, of which 29 were assigned to the high GI and 29 to the low GI diet (per protocol results). Twelve subjects discontinued because they were unwilling to adhere to the diet (four on the low GI diet and eight on the high GI diet) and three subjects were omitted from data analysis due to missing acne gradings (dermatologist appointments were cancelled due to illness). Forty-three subjects completed the study (n = 23 low GI and n = 20 high GI). The mean ± SD age (16.6 ± 1.1 y) and BMI (23.6 ± 3.8 kg/m^2^) of the completers was not significantly different to the subjects who discontinued (age 16.4 ± 0.7 y and BMI 21.9 ± 2.3 kg/m^2^, p = 0.72 and p = 0.12, respectively). On the whole, there were no significant differences in subject characteristics, baseline acne severity or blood biochemistry between the two diet groups (Table 1). However, at baseline, DHEA-S was higher in those allocated to the low GI diet (p = 0.04). As determined by the study protocol, diets differed significantly in GI (low GI diet 51 ± 1 *vs.* high GI diet 61 ± 2, p = 0.0002), but not energy, protein, total fat, carbohydrate, fiber, zinc or calcium content (Table 2). Diets also differed significantly in saturated fat (low GI diet 13 ± 0% *vs.* high GI diet 15 ± 1%, p = 0.01). Changes in weight were minor and not different within or between diet groups (low GI −0.3 ± 0.4 kg *vs.* high GI 1.1 ± 0.6 kg, p = 0.053).

**Table 1:** Baseline subject characteristics, acne severity and blood variables.

	Low GI, n = 23	High GI, n = 20	P
	**Mean ± SEM**	**Mean ± SEM**	
**Age (y)**	16.6 ± 0.2	16.5 ± 0.3	0.92
**BMI (kg/m^2^)**	24.0 ± 1.0 ^a^	23.1 ± 0.6 ^b^	0.45
**Weight (kg)**	76.0 ± 3.3	75.4 ± 1.8	0.87
**Facial acne score/3**	2.1 ± 0.1	1.9 ± 0.2	0.30
**Glucose (mmol/L)**	4.7 ± 0.1 ^c^	4.8 ± 0.1	0.54
**Insulin (pmol/L)**	22.7 ± 4.1 ^c^	22.9 ± 2.3	0.98
**HOMA-IR**	0.7 ± 0.1 ^c^	0.8 ± 0.1	0.51
**Testosterone (nmol/L)**	34.3 ± 1.8 ^c^	32.4 ± 1.6	0.43
**SHBG (nmol/L)**	23.7 ± 2.4 ^c^	26.4 ± 2.1	0.59
**FAI (nmol/L)**	177.7 ± 20.6 ^c^	142.6 ± 12.7	0.16
**DHEA-S (µmol/L)**	**5.9 ± 0.6 ^c^**	**4.5 ± 0.4**	**0.04**
**IGF-1 (nmol/L)**	56.6 ± 4.7 ^c^	57.8 ± 3.6	0.84
**IGFBP-1 (ng/mL)**	33.4 ± 6.0 ^c^	30.8 ± 3.1	0.71
**IGFBP-3 (µg/mL)**	6.2 ± 0.3 ^c^	6.3 ± 0.4	0.84

DHEA-S: dehydroepiandrosterone-sulfate; FAI: free androgen index; GI: glycemic index; HOMA‑IR: homeostasis modeling assessment of insulin resistance; IGF-1: insulin-like growth factor-1; IGFBP-1 and -3: insulin like growth factor binding proteins -1 and -3; P: independent samples t-test; SHBG: sex hormone binding globulin; ^a^: n = 22; ^b^: n = 19; ^c^: n = 20; Three subjects on the low GI diet refused blood collection.

**Table 2:** Daily nutrient intakes calculated from the food diaries that were filled out every weekend during the intervention period for eight weeks.

	Low GI, n = 20 ^a^	High GI, n = 17 ^b^	p
	**Mean ± SEM**	**Mean ± SEM**	
**E (kJ)**	8164 ± 584	9417 ± 571	0.14
**Protein (%E)**	21 ± 1	19 ± 1	0.06
**Fat (%E)**	34 ± 1	36 ± 1	0.11
**Monounsaturated fat (%E)**	13 ± 0	13 ± 0	0.46
**Polyunsaturated fat (%E)**	4 ± 0	4 ± 0	0.85
**Saturated fat (%E)**	**13 ± 0**	**15 ± 1**	**0.01**
**Carbohydrate (%E)**	39 ± 2	42 ± 2	0.25
**Fiber (g)**	16 ± 2	15 ± 1	0.5
**Zinc (mg)**	13 ± 1	14 ± 1	0.44
**Calcium (mg)**	640 ± 67	796 ± 83	0.15
**Glycemic index**	**51 ± 1**	**61 ± 2**	**0.0002**
**Glycemic load**	**102 ± 9**	**157 ± 18**	**0.01**

E: energy; ^a^: 3 subjects from the low GI diet lost their food diaries; ^b^: 3 subjects from the high GI diet lost their food diaries.

**Figure 1:**
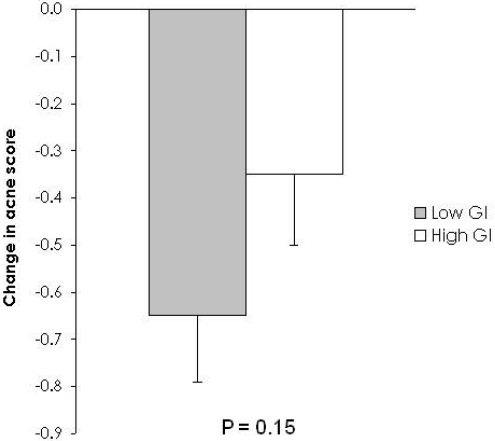
Change in facial acne severity from week 0 to week 8. Facial acne severity decreased more on the low glycemic index (GI) diet, but the difference between diets did not reach significance (acne score change: low GI mean ± SEM, n = 23, −0.65 ± 0.14 *vs.* high GI mean ± SEM, n = 20, −0.35 ± 0.15, p = 0.15).

Facial acne improved on both diets (low GI −26 ± 6%, p = 0.0004 and high GI −16 ± 7%, p = 0.01), but differences between diets did not reach significance (Figure 1, Table 3). Similarly, there were no significant differences within or between diets for most biochemical parameters. However, IGFBP-1 declined significantly on the low GI diet *versus* the high GI diet (low GI −6.2 ± 3.7 ng/mL *vs.* high GI 5.0 ± 2.6 ng/mL, p = 0.02) (Table 4). Testosterone increased significantly from 0 to 8 wk on the high GI diet and increased non-significantly on the low GI diet, but there was no effect of diet composition (low GI 3.4 ± 1.8 nmol/L *vs.* high GI 5.0 ± 1.7 nmol/L, p = 0.50).

Change in acne severity was not correlated with change in HOMA-IR or change in insulin (Pearson correlation r = −0.196, p = 0.244 and Pearson correlation r = −0.202, p = 0.225, respectively). 

In the present study we found no effect of GI on facial acne severity over 8 wk and no correlation between changes in acne severity and insulin concentrations or sensitivity. However, our hypothesis that a low GI diet would improve facial acne to a greater extent than a high GI diet was consistent with the finding that acne severity decreased more on the low GI diet (~26%, p = 0.0004) compared with the high GI diet (~16%, p = 0.01). Because the trial duration was dictated by the school term, our study was shorter than the ideal and usual intervention period of 12 wk in acne treatment studies [2,17]. 

**Table 3:** Change in acne severity from week 0 to week 8 adjusted for differences in dermatologist, grading method and baseline ^1^.

	Diet	Mean ± SEM	p ^2^	Covariate and (p value) ^3^
Facial acne ^4^	Low GI	−0.65 ± 0.14	0.15	
	High GI	−0.35 ± 0.15		
Facial acne ^5^	Low GI	−0.61 ± 0.13	0.28	−0.42 (0.004)
	High GI	−0.40 ± 0.14		

GI: glycemic index; ^1^ Low GI diet, n = 23, high GI diet, n = 20; ^2^ p value between groups (adjusted for differences in change in dermatologist and photo/in person acne grading method, but not adjusted for differences in baseline); ^3^ Coefficient of covariate (p value) for results that have been adjusted for differences in baseline; ^4^ Change in acne severity mean values adjusted for differences in change in dermatologist and photo/in person acne grading method, but not adjusted for differences in baseline; ^5^ Change in acne severity mean values adjusted for differences in change in dermatologist and photo/in person acne grading method and adjusted for differences in baseline.

**Table 4:** Change in blood parameters from week 0 to week 8 ^1^.

	Diet	Mean ± SEM	p ^2^	p ^3^
**Glucose (mmol/L)**	Low GI	−0.1 ± 0.1	0.58	0.74
	High GI	−0.1 ± 0.1	0.32	
**Insulin (pmol/L)**	Low GI	5.5 ± 3.0	0.09	0.49
	High GI	2.3 ± 3.2	0.27	
**HOMA-IR**	Low GI	0.2 ± 0.1	0.10	0.60
	High GI	0.1 ± 0.1	0.39	
**Testosterone (nmol/L)**	Low GI	3.4 ± 1.8	0.07	0.50
	High GI	5.0 ± 1.7	0.03	
**SHBG (nmol/L)**	Low GI	0.4 ± 1.2	0.71	0.75
	High GI	−0.1 ± 1.0	0.81	
**FAI (nmol/L)**	Low GI	14.4 ± 10.3	0.17	0.92
	High GI	11.4 ± 7.9	0.16	
**DHEA-S (µmol/L)**	Low GI	0.2 ± 0.2	0.35	0.86
	High GI	0.3 ± 0.2	0.17	
**IGF-1 (nmol/L)**	Low GI	1.8 ± 1.9	0.35	0.10
	High GI	1.9 ± 2.4	0.43	
**IGFBP-1 (ng/mL)**	Low GI	−6.2 ± 3.7	0.11	0.02
	High GI	4.0 ± 2.6	0.14	
**IGFBP-3 (µg/mL)**	Low GI	−0.3 ± 0.4	0.46	0.86
	High GI	−0.1 ± 0.2	0.58	

DHEA-S: dehydroepiandrosterone-sulfate; FAI: free androgen index; GI: glycemic index; HOMA‑IR: homeostasis modeling assessment of insulin resistance; IGF-1: insulin-like growth factor-1; IGFBP-1 and -3: insulin like growth factor binding proteins -1 and -3; SHBG: sex hormone binding globulin; ^1^ Low GI diet, n = 19, high GI diet, n = 19 (5 subjects, 4 in low GI diet and 1 in high GI diet, did not provide blood samples); ^2^ p valuewithin groups from 0 to 8weeks; ^3^ p value between groups.

The mean change in acne severity on the low GI diet was a decrease of over half a point out of three, which represents a change that is likely to be highly significant to subjects [18]. More sensitive and objective dermatological assessment tools may have highlighted differences to a greater extent. 

Improvements in acne on both the low and the high GI diet occurred despite the concurrent increase in testosterone levels, which reached significance on the high GI diet. Androgen levels increase during adolescence, hence higher testosterone may reflect a period of rapid sexual maturation [9,19]. In practice, androgen concentrations have been directly correlated to the severity of acne [20,21], thus the increases in testosterone might be expected to increase acne severity.

The findings of the present study contrast with those of Smith *et al.* [2]. Those investigators managed a 13 point difference in GI (43 *vs.* 56), and GI values were lower in both groups, whereas the current study produced a 10 point difference in GI (51 *vs.* 61). However, differences in GL were similar because we had slightly lower intakes of carbohydrate (Smith *et al.* [2] 101 *vs.* 174; current 102 *vs.* 157). Saturated fat was significantly different between interventions in both studies, which could have affected results because dietary fats can directly enter the pilosebaceous unit from the blood [22]. Recent evidence suggests that an increased proportion of saturated fatty acids in skin surface triglycerides may decrease abnormal skin cell replication and improve acne [23,24].

Overall, our findings imply that low GI carbohydrates were not responsible for the improvements in facial acne reported by Smith *et al.* [2]. The increased weight loss on the low GL arm of that study (nearly 3 kg compared to the weight gain of 0.5 kg in the control group) suggests negative energy balance and could explain the declines in fasting insulin and HOMA-IR. The higher protein content and higher fiber of their low GL diet may have contributed to increased satiety and reduced food intake [25,26]. Energy restriction and weight loss *per se* are recognized to improve fasting insulin concentration and HOMA-IR, independent of diet composition. Even in the absence of weight loss, higher protein or fiber intake may lead to improvements in glucose and insulin metabolism [27,28]. SHBG production also increases after weight loss, implying decreased androgenic activity [8]. Indeed, when Smith *et al.* adjusted for change in BMI, the differences in total inflammatory plus non-inflammatory acne lesion counts, HOMA-IR, SHBG and FAI between diets were no longer significant (although IGFBP-1 and inflammatory lesion counts remained significant) [2].

We found a significant effect of diet on IGFBP-1 but not IGFBP-3 or IGF-1. However, unlike Smith *et al.*, IGFBP-1 decreased on the low GI diet [2].In an acute study in lean, young healthy subjects, Brand-Miller *et al.* observed that serum IGFBP-1 declined acutely after both high and low GI meals; the mean change at 4 h being significantly more prolonged after the low GI meal [29]. This is consistent with the decrease in IGFBP-1 in the present study. Considering the improvement in acne within both diets, we might have expected IGFBP-1 to increase on both diets and IGF-1 to decrease [10,21]. It is possible that blood levels of IGF-1 do not directly reflect levels in cells and tissues and that the complexity of IGF and IGFBP interactions and dietary effects on the system makes interpretation difficult.

Unlike Smith *et al.*, we failed to see changes in SHBG or DHEA-S [2]. Improvements in acne severity might be expected to correspond to an increase in SHBG [30] and a fall in DHEA-S [21,31]. In postmenopausal women with hyperandrogenism, a low GI diet was able to increase SHBG and decrease testosterone concentration, although the study duration was longer [32]. Some studies have reported no association between free testosterone and acne severity [31], potentially because measurement of testosterone is prone to error. It is removed quickly from the circulation by the liver and target tissues, and may be produced locally in the pilosebaceous intracrine system [20].

The limitations of our study should be noted. Twelve subjects discontinued the study and three subjects were not available for the final acne gradings, representing a substantial drop out rate (26%). The 4-point acne grading method was novel and hence not internationally validated and capable of detecting only relatively large changes in the severity of inflammatory lesions (a change of one grade or more, e.g., from severe to moderate acne). Less obvious changes in inflammatory and non‑inflammatory lesions may have been overlooked. Assessing acne severity at four weeks would have increased the scope of the study, but this was not possible due to commitments of the dermatologist. Studying adolescents at boarding school may prevent generalization of results to wider non-boarding adolescent population. The non-randomized alternate allocation study design may have resulted in selection bias. A control group (who were not given any dietary advice) would have been useful to determine the true significance of within-diet changes. The subjects were not blinded to the purpose of the study and food intake may have been under- or over-reported. Changes in insulin sensitivity may have occurred but gone undetected because HOMA-IR is a comparatively “blunt” measure of insulin sensitivity, reflecting hepatic rather than peripheral glucose metabolism. Food intake was not measured at baseline, so differences between habitual diet and intervention diets could not be assessed. Food diaries were only completed on weekends because it was felt that asking the students to record their food intake in such detail for a longer period would be too onerous, which meant that any differences between week day and weekend food intakes would have been missed (although the vast majority of students remained at school on weekends and hence week day diet would be similar to weekend diet). Validation of the food diaries (e.g., via weighed food records) may have increased the accuracy of the intake data. Finally, the difference in GI between diets may not have been large enough to materially affect postprandial glycemic and insulin responses in adolescent males. Larger differences (ranging from 13 to 28 units) were employed to effect changes in glycated proteins in diabetic subjects [33]. The GI of our high GI intervention may have been similar to habitual intake. 

A myriad of emotional and physiologic factors affect the pathogenesis of acne, making dietary studies in this area especially difficult, including stress [34,35] and nutrients other than carbohydrates (saturated fats [22,24], n-3 *vs.* n-6LCPUFA [36,37,38,39], vitamin D [40] and dairy [41]). n-3LCPUFA are increasingly recognized as anti-inflammatory agents [36], while n-6 fats are precursors of highly active mediators of inflammation [37]. n-3LCPUFA have been shown to increase IGFBP-3 in animals [38] and decrease IGF-1 in humans [39]. In this study, we found no difference in LCPUFA between diets, but did not differentiate between n-3 and n-6 isoforms.

## 4. Conclusions

This study suggests that a low GI diet does not significantly improve facial acne more than a macronutrient-matched high GI diet, although there was a trend for the low GI diet to improve acne to a greater extent than the high GI diet. However, the limitations of the study limit the conclusions that can be made from the results. The lack of a difference between diets may be due to short study duration and a relatively blunt acne severity assessment tool. It is possible that the observations of the previous study investigating dietary GI/GL may be explained by weight loss or differences in protein and/or fiber content. Future studies should employ randomized designs of at least 12 wk duration, subjects within a narrow age range, and using validated acne scoring systems [17]. Tight control of confounding factors, including weight, type and amount of fat, protein and carbohydrate, level of stress, and other limitations discussed above, is necessary.
